# Exploratory Analysis of Qualitative MR Imaging Features for the Differentiation of Glioblastoma and Brain Metastases

**DOI:** 10.3389/fonc.2020.581037

**Published:** 2020-12-10

**Authors:** Raphael Meier, Aurélie Pahud de Mortanges, Roland Wiest, Urspeter Knecht

**Affiliations:** ^1^University Institute of Diagnostic and Interventional Neuroradiology, University Hospital Bern, Inselspital, University of Bern, Bern, Switzerland; ^2^Support Center for Advanced Neuroimaging, University Hospital Bern, Inselspital, University of Bern, Bern, Switzerland; ^3^ARTORG Center for Biomedical Research, University of Bern, Bern, Switzerland; ^4^Department of Diagnostic Radiology and Neuroradiology, Regional Hospital Emmental, Burgdorf, Switzerland

**Keywords:** qualitative magnetic resonance features, Visually AcceSIble Rembrandt Images, exploratory data analysis, differentiation, glioblastoma, brain metastasis, machine learning, support vector machines

## Abstract

**Objectives:**

To identify qualitative VASARI (Visually AcceSIble Rembrandt Images) Magnetic Resonance (MR) Imaging features for differentiation of glioblastoma (GBM) and brain metastasis (BM) of different primary tumors.

**Materials and Methods:**

T1-weighted pre- and post-contrast, T2-weighted, and T2-weighted, fluid attenuated inversion recovery (FLAIR) MR images of a total of 239 lesions from 109 patients with either GBM or BM (breast cancer, non-small cell (NSCLC) adenocarcinoma, NSCLC squamous cell carcinoma, small-cell lung cancer (SCLC)) were included. A set of adapted, qualitative VASARI MR features describing tumor appearance and location was scored (binary; 1 = presence of feature, 0 = absence of feature). Exploratory data analysis was performed on binary scores using a combination of descriptive statistics (proportions with 95% binomial confidence intervals), unsupervised methods and supervised methods including multivariate feature ranking using either repeated fitting or recursive feature elimination with Support Vector Machines (SVMs).

**Results:**

GBMs were found to involve all lobes of the cerebrum with a fronto-occipital gradient, often affected the corpus callosum (32.4%, 95% CI 19.1–49.2), and showed a strong preference for the right hemisphere (79.4%, 95% CI 63.2–89.7). BMs occurred most frequently in the frontal lobe (35.1%, 95% CI 28.9–41.9) and cerebellum (28.3%, 95% CI 22.6–34.8). The appearance of GBMs was characterized by preference for well-defined non-enhancing tumor margin (100%, 89.8–100), ependymal extension (52.9%, 36.7–68.5) and substantially less enhancing foci than BMs (44.1%, 28.9–60.6 *vs.* 75.1%, 68.8–80.5). Unsupervised and supervised analyses showed that GBMs are distinctively different from BMs and that this difference is driven by definition of non-enhancing tumor margin, ependymal extension and features describing laterality. Differentiation of histological subtypes of BMs was driven by the presence of well-defined enhancing and non-enhancing tumor margins and localization in the vision center. SVM models with optimal hyperparameters led to weighted F1-score of 0.865 for differentiation of GBMs from BMs and weighted F1-score of 0.326 for differentiation of BM subtypes.

**Conclusion:**

VASARI MR imaging features related to definition of non-enhancing margin, ependymal extension, and tumor localization may serve as potential imaging biomarkers to differentiate GBMs from BMs.

## Introduction

Brain metastases (BMs) are the most common tumors of the central nervous system (1, 2). With an incidence of about 5,000 newly diagnosed cases every year in Switzerland (3), they by far exceed primary brain tumors (around 600 newly diagnosed cases per year) (4). Among primary brain tumors, glioblastoma multiforme (GBM), a very malignant form of diffusely infiltrating WHO grade IV astrocytoma, is the most frequent in adults (5).

BMs and GBMs have different growth patterns on a cellular scale, with BMs usually presenting as well-defined spherical lesions, which displace adjacent brain tissue without notable infiltration (6) and GBMs mostly exhibiting invasive growth patterns with infiltration of the surrounding structures, favorably white matter tracts (7). Nevertheless, they may be hard to distinguish on MR images, if no evident features such as multiplicity for BM or transhemispheric spread for GBM is present (8).

The morphology of brain tumors can be characterized in different ways, for example through volumetric segmentation of the tumor compartments (9), *i.e.* quantitative analysis, or by characterization of qualitative features. Quantitative analysis notably also includes the analysis of heterogeneity of brain tumors which has been demonstrated to be an important imaging biomarker for differentiation of cancerous tissues in gliomas (10–12). For primary brain tumors a set of qualitative features has been defined by a group of experienced neuroradiologists from the cancer research community to enable standardized scoring of subjective MR features, which are regularly encountered on routine contrast-enhanced MR images. This set is called the VASARI feature guide [*V*isually *A*cces*SI*ble *R*EMBRANDT (The Repository of Molecular Brain Neoplasia Data) *I*mages], and it currently comprises 24 morphologic features, describing the location of the tumor, characteristics of the tumor compartments, and presence of distinct features such as hemorrhage or pial invasion (13–15). Even before VASARI, researchers assessed the utility of such information (16). For example already in 2005, Pope et al. studied the relationship between 15 imaging variables and survival in patients with grade III/IV gliomas (17), but the VASARI feature guide allows radiologists to study and report their findings in a standardized way, largely independent of the rater, institution, and approach used (16).

The VASARI features have been employed for different research questions, most commonly prediction of patient survival (18–20) or prediction of tumor progression (21). Most of these studies did not evaluate the predictive quality of VASARI features alone, but in combination with clinical, pathological and/or molecular data. Some used the TCGA (*T*he *C*ancer *G*enome *A*tlas)-GBM dataset (13, 19, 22–24) as it provides easily accessible and ready to use imaging data including a variety of additional information about the available patients (25).

Even though most commonly used to describe GBMs, the VASARI features have also been applied to lower grade gliomas (LGG): Hyare et al. tried to predict isocitrate dehydrogenase 1 (IDH1) mutation status (26), Zhou et al. aimed at predicting histological grade and tumor progression as well as mutation status (IDH1 and 1p/19q codeletion) (27) and Lehrer et al. evaluated the relationship between MR tumor characteristics and protein measurements (28).

So far, the VASARI feature set has only been applied to primary brain tumors; its use in brain metastases has not been evaluated. Consequently, this study explores the applicability of the VASARI feature set in patients with BMs. The objective is the identification of a subset of VASARI features for the diagnostic discrimination among GBMs and BMs of different primary tumors.

## Materials and Methods

### Study Population

Eligible for this study were patients admitted to the Inselspital Bern between 2000 and 2018 with histologically confirmed diagnosis of one of the following five brain pathologies: 1) brain metastasis (BM) from carcinoma of the breast, 2) BM of non-small cell (NSCLC) adenocarcinoma of the lung, 3) BM NSCLC squamous cell carcinoma (SCC) of the lung, 4) small cell carcinoma of the lung (SCLC), or 5) GBM. The collective of eligible patients was reviewed for existence of pre-operative MR images.

**Figure 1:**
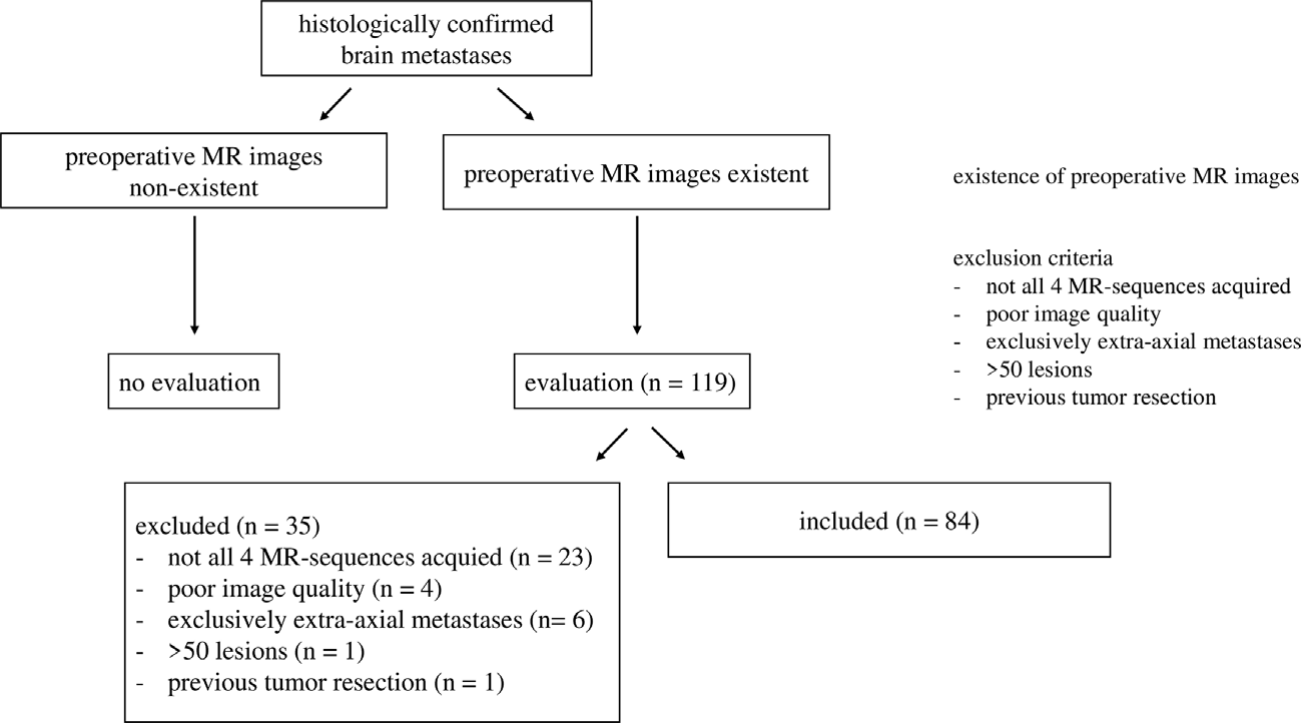
Inclusion of patients with brain metastases. The final number of included patients is n = 84.

Initially, 119 patients with histologically confirmed BMs were included. For the BM groups, exclusion occurred upon: one or more of the four required MR sequences are unavailable (n = 23, detailed in Section *Imaging Protocol*), poor image quality (n = 4), previous tumor resection (n = 1), exclusively extra-axial lesions (n = 6), and unmanageable number of metastases (>50, n = 1) (*cf*. **Figure 1**). For every BM group we included all patients meeting the inclusion and exclusion criteria, up to a maximum of 30 patients per group. In groups with more than 30 patients meeting the criteria we gave preference to the ones who underwent imaging more recently due to better imaging quality in recent years. We were able to include a total of 84 BM patients: 30 with BM from carcinoma of the breast, 13 with BM from NSCLC SCC of the lung, 30 with BM from NSCLC adenocarcinoma of the lung, and 11 with BM from SCLC.

25 GBM patients were included from a patient cohort published previously in context of brain tumor segmentation (29). Lesions which did not show any enhancing tumor component or exhibited a very large proportion of non-enhancing tumor were not considered in our analysis (in total five lesions in four patients).

This led to a total of 109 included patients (84 with BM, 25 with GBM). The research described in this paper took place at the Inselspital Bern, in the context of the trial *CATCh*, a single-center retrospective cohort study without intervention, using MR images which have been acquired in the process of clinical diagnostics. *CATCh* has been approved by the local research ethics commission (Kantonale Ethikkomission Bern).

### Imaging Protocol

Due to the extensive time span of patient eligibility and images being partly externally acquired, imaging protocols for BMs were highly heterogeneous (parameter values are reported as ranges). MR images were acquired on 1.5 or 3 T MR scanners from Philips Medical Systems, Siemens and GE Medical Systems. Four representative MR sequences were used: T2-weighted (T2), T2-weighted with fluid attenuated inversion recovery (FLAIR), native T1-weighted (T1) and T1-weighted with gadolinium contrast-agent (T1c). Sequence parameters: T2) acquired as a T2 SPACE iso-voxel sequence with a slice thickness of 1 mm in sagittal direction or as spin-echo or turbo spin-echo sequence with a slice thickness of 3–6 mm in axial direction, using an echo time (TE) of 13–409 ms and a repetition time (TR) of 438–15,000 ms. FLAIR) acquired as T2 SPACE dark fluid iso-voxel sequence with slice thickness 0.9 mm or 1.4 mm in sagittal direction or as FLAIR-sequence with slice thickness 3–6 mm in coronary or axial direction, TE 7.4–386 ms, TR 2,000–11,000 ms. T1) acquired as gradient echo sequence with a slice thickness of 1 mm in sagittal direction or as spin-echo sequence with a slice thickness of 3–6 mm in axial direction, TE 1.5–17 ms, TR 164–1,910 ms. T1c) acquired as gradient echo sequence with a slice thickness of 0.9 or 1 mm in sagittal direction, T1 vibe iso-voxel sequence with a slice thickness of 0.8 or 0.9 mm in transversal direction or as spin-echo sequence with a slice thickness of 3–6 mm in axial direction, with gadolinium enhancement, TE 2.3–17 ms, TR 6.1–2,320 ms.

For the GBMs, a standardized MR protocol was used for all patients. All sequences were acquired on a 1.5 T MR scanner from Siemens (Siemens Avanto and Siemens Area, Siemens, Erlangen/Germany). The protocol included T2) acquired as a 3D T2w SPACE in sagittal direction, TE 380 ms, TR 3000 ms, FOV 256 × 256 mm^2^, FA 120°, isotropic voxel size of 1 mm × 1 mm × 1 mm. FLAIR) acquired as a 2D T2w FLAIR in axial direction, TE 80 ms, TR 8,000 ms, FOV 256 × 256 mm^2^, FA 120°, anisotropic voxel size of 1 mm × 1 mm × 3 mm. T1) acquired as native 3D T1w MPR in sagittal direction, TE 2.67 ms, TR 1580 ms, FOV 256 × 256 mm^2^, FA 8°, isotropic voxel size of 1 mm × 1 mm × 1 mm. T1c) acquired as a 3D T1w sequence with gadolinium contrast enhancement in sagittal acquisition, TE 4.57 ms, TR 2070 ms, FOV 256 × 256 mm^2^, FA 15°, isotropic voxel size of 1 mm × 1 mm × 1 mm.

### Visually AcceSIble Rembrandt Image Magnetic Resonance Features

Based on the VASARI MR feature guide, we derived a set of morphological features which were evaluated for a total of 239 individual brain lesions from 109 patients. The defined set comprised localization-based features as well as appearance-based features.

The localization-based features included the involvement of

-left/right hemisphere or central structure
-cerebral lobes: frontal, parietal, temporal and occipital lobe
-insular cortex
-subcortical structures: basal ganglia, thalamus, brainstem and Corpus callosum
-cerebellum
-eloquent brain areas: vision center (area around Sulcus calcarinus), auditory center (Gyri temporales transversi), Wernicke’s area (from dorsal region of Gyrus temporalis superior to the Gyri angularis et supramarginalis of the parietal lobe), Broca’s area (Partes triangularis et opercularis of Gyrus frontalis inferior), primary somatosensory cortex (Gyrus postcentralis) and primary somatomotor cortex (Gyrus praecentralis).

The localization-based features were not treated as mutually exclusive, but every lesion was attributed to multiple of the above-mentioned categories, *e.g.* right hemisphere, temporal lobe, vision center.

The appearance-based features included

-definition of contrast-enhancing margin (margin of contrast-enhancing tumor compartment (CET), strongly T1c hyperintense) and non-enhancing margin (margin of the non-contrast enhancing tumor compartment [nCET]/peritumoral edema, FLAIR/T2 hyperintense)
-existence of: hemorrhage, which was defined as T1 hyperintensity visible in both T1 native and T1c images, pial invasion, ependymal invasion
-involvement of cortex
-crossing of brain midline by the CET and nCET
-multiplicity of enhancing foci.

We chose these features from the VASARI guide by excluding semi-quantitative measurements (*i.e.* proportion of compartment 1 to compartment 2) and features that evaluate post-interventional status, as we *a priori* excluded patients who already underwent surgical treatment. Furthermore, very rarely observed features in GBMs and BMs such as calvarian remodeling have been omitted.

In order to facilitate the rating of VASARI MR features, the four MR sequences (T1, T1c, T2, FLAIR) were rigidly co-registered using a versor 3D rigid transform optimized using Mattes Mutual Information metric from the Insight Toolkit (ITK) (30). The feature evaluation for all 239 lesions has been consecutively performed by a medical student (AP) and was subsequently confirmed by an experienced, board-certified neuroradiologist with more than 8 years of experience in brain tumor diagnostics (UPK).

### Statistical Analysis

The aim of the exploratory data analysis was to identify patterns in the data, which could serve as a basis for hypothesis formulation and subsequent prospective confirmatory analysis. The VASARI features correspond to asymmetric binary attributes (1 = presence or 0 = absence of a feature). The presence of features for two lesions implies that they are more similar to one another, while the absence of features does not carry the same amount of information. Thus, the VASARI features are considered as asymmetric binary attributes. In the first phase, the features were counted, and proportions were computed for each histological type separately. Proportions were visualized using heatmaps, and 95% binomial confidence intervals for proportions were estimated using the Wilson method (31). In the second phase, the localization-based and appearance-based MR features were combined for an unsupervised exploratory data analysis. Pairwise differences were measured among binary feature vectors of all lesions and histological subtypes using the Jaccard distance (=1 − Jaccard index). The resulting distance matrix (of dimension 239 × 239) was clustered using agglomerative hierarchical clustering with average linkage. Furthermore, pairwise differences were summarized across all lesions (by averaging) within a given histological tumor type, to yield a distance matrix for the different types. The resulting distance matrix was transformed to an affinity matrix of an undirected graph by computing its entries a_i,j_ = 1 − d_i,j_ with d_i,j_ being the (i,j)^th^-entry of the distance matrix. Finally, the undirected graph is visualized using a spectral layout, which puts nodes with high affinity closer to each other than nodes with low affinity. This procedure was repeated for different VASARI feature subsets. Subsets were generated by exclusion of features if they exhibited proportions of less than X% across all histological tumor types (with X ranging from 15 to 90%, in 15% increments). The rationale for the exclusion is to remove features which do not carry sufficient information for the purpose of tumor type differentiation. In the third phase, the localization-based and appearance-based MR features were combined for a supervised exploratory data analysis, which included the tumor class label in the computation. Differentiation of BMs from GBMs was formulated as a binary classification problem and differentiation among histological subtypes of BMs as a multi-class problem (four classes). Univariate feature ranking was performed using the Mutual Information between feature values and tumor class labels. Multivariate feature ranking was performed by repeatedly fitting a linear soft-margin Support Vector Machine (SVM) to the data. In each iteration, the hyperparameter C [with values (0.001, 0.01, 0.1, 1, 10, 100)] and/or class balancing (on/off) was changed, resulting in a total of 12 models. Stratified 3-fold cross-validation with weighted-F1-score as performance metric was performed to find the optimal hyperparameter setting for the binary discrimination of BMs *versus* GBMs and for the multi-class problem of discriminating the histological subtypes of BMs. The latter was implemented in a one-*vs*-all approach. Finally, based on the optimal hyperparameters, feature ranking by recursive feature elimination was performed as an additional multivariate method. Descriptive statistics were computed using R (version 4.0.0) (32); unsupervised and supervised feature analyses were implemented using Python’s networkx (version 2.4) and scikit-learn (version 0.22.1) modules.

## Results

### Patient Characteristics

**Figure 2:**
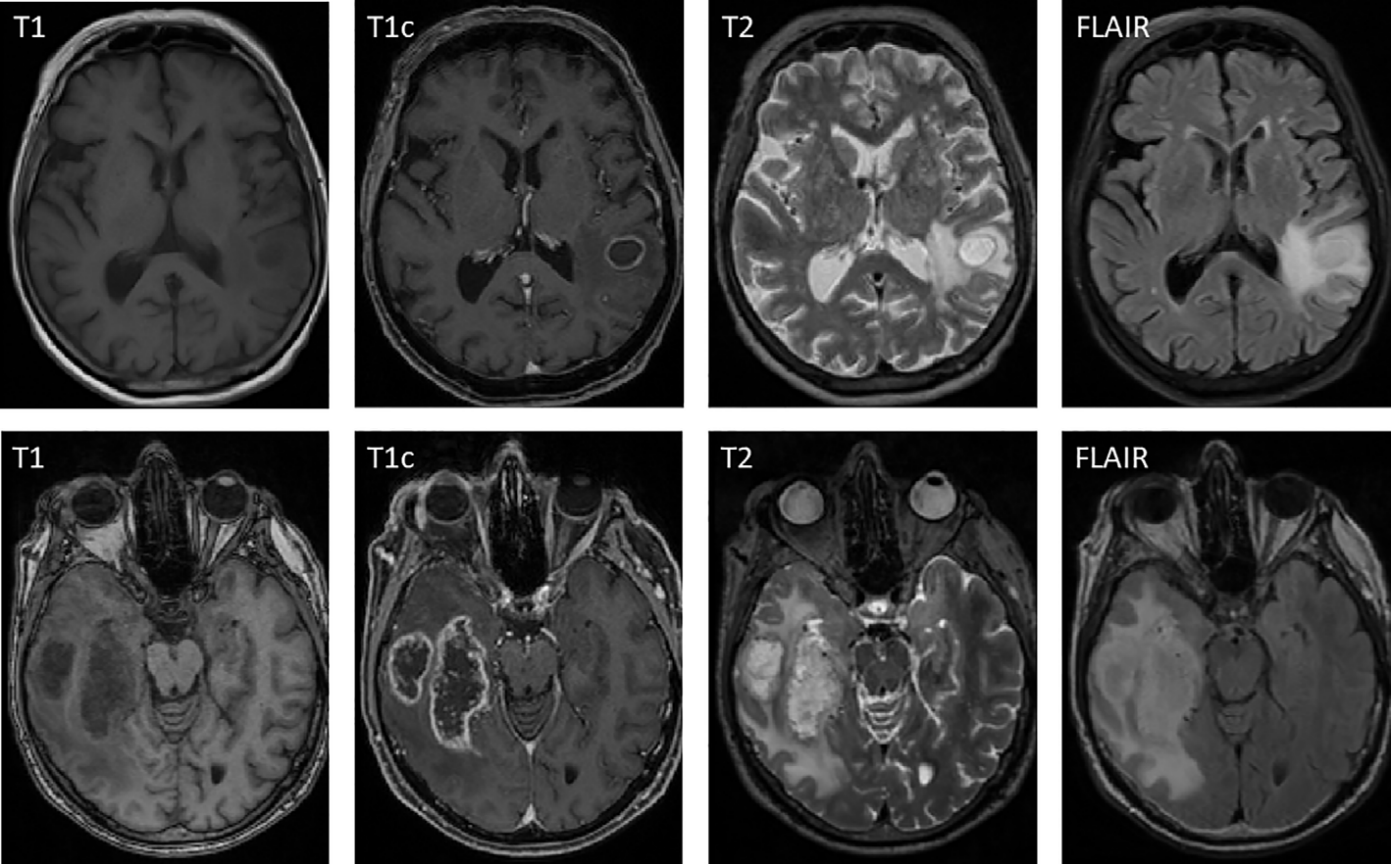
Exemplary case of a solitary brain metastasis from a NSCLC adenocarcinoma (top row) shown alongside an exemplary glioblastoma case (bottom row). In both cases, contrast-enhancing tumor, central necrosis, and peritumoral edema are visible.

Among the 109 patients included in this study are 63 women and 46 men. This asymmetry is explained by the fact that there are only women in the breast cancer group. According to the Swiss Cancer League, there have been only around 50 cases of breast cancer in men per year in the period of 2012–2016 (33). NSCLC SCC presented with an asymmetry as well, occurring in 10 men and three women. The median age for all groups is 63.37 years, with a minimum of 27.13 years (GBM) and a maximum of 81.92 years (breast cancer). For more detailed information, see **Table 1**. In **Figure 2**, an exemplary BM of a NSCLC adenocarcinoma is shown alongside an exemplary GBM case. Evidently both BM and GBM can exhibit contrast-enhancing tumor, central necrosis, and peritumoral edema.

**Table 1:** Characteristics of included patients (n = 109) with five different histological tumor types (breast cancer, NSCLC Adenocarcinoma, NSCLC SCC, SCLC, GBM).

Feature	Breast cancer n = 30	NSCLC Adeno n = 30	NSCLC SCC n = 13	SCLC n = 11	GBM n = 25
Male Female	0 (0) 30 (100%)	16 (53%) 14 (47%)	10 (77%) 3 (23%)	7 (64%) 4 (36%)	13 (52%) 12 (48%)
Age (median, IQR)	53.26 (46.35, 60.68)	62.27 (54.88, 65.91)	64.88 (63.09, 69.48)	63.37 (60.69, 65.10)	69.46 (65.09, 73.13)
Number of lesions	93	61	18	33	34

### Localization-Based Magnetic Resonance Features

#### Glioblastoma *Versus* Metastasis

**Figure 3:**
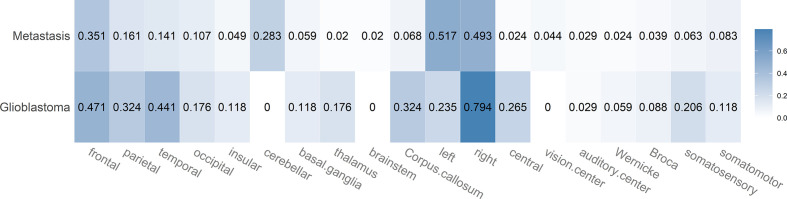
Heatmap showing the proportions for the different localization-based MR features in case of brain metastases and glioblastoma. Each proportion corresponds to the ratio: number of lesions with feature/total number of lesions of that class. Darker color indicates a larger proportion.

The proportions of localization-based MR features for GBMs and BMs are shown in **Figure 3**. Whereas brain metastases seemed equally distributed over both brain hemispheres (51.7%, 95% CI 44.9–58.5 *vs.* 49.3%, 95% CI 42.5–56.1), it appeared that GBMs tend to be localized more often in the right hemisphere (79.4%, 95% CI 63.2–89.7). They also affected central structures more than brain metastases did, especially the thalamus (17.6%, 95% CI 8.3–33.5) and corpus callosum (32.4%, 95% CI 19.1–49.2). We did not observe any cases where GBM appeared infratentorially (in the cerebellum or brainstem). GBMs involved all lobes of the cerebrum, though with a slight fronto-occipital gradient, the frontal lobe being affected most frequently (47.1%, 95% CI 31.5–63.3) and the occipital lobe least frequently (17.6%, 95% CI 8.3–33.5). The somatosensory cortex (Gyrus postcentralis) was infiltrated by GBMs rather frequently (20.6%, 95% CI 10.3–36.8); no case of infiltration of the primary visual cortex (area around Sulcus calcarinus) was observed. BMs showed a preference for localization in the frontal lobe (35.1%, 95% CI 28.9–41.9) and cerebellum (28.3%, 95% CI 22.6–34.8).

#### Comparison of Histological Subtypes

**Figure 4:**
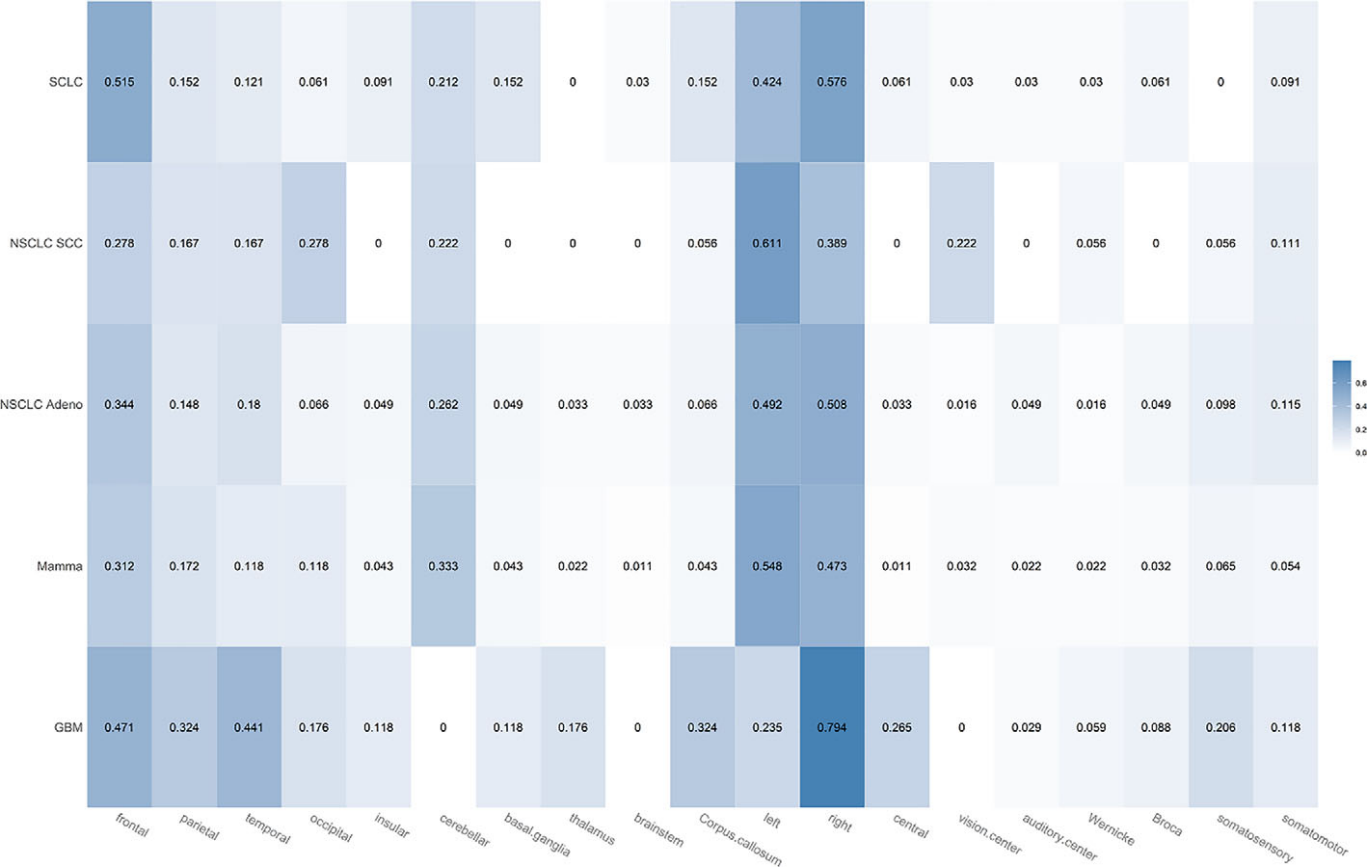
Heatmap showing the proportions for the different localization-based MR features broken down for the different histological subtypes of brain metastases (SCLC, NSCLC SCC, NSCLC adenocarcinoma, mammacarcinoma). Each proportion corresponds to the ratio: number of lesions with feature/total number of lesions of that class. Darker color indicates a larger proportion.

In **Figure 4**, the proportions of localization-based MR features are broken down for the different histological subtypes of BMs. BMs from breast cancer occurred more frequently in the cerebellum (33.3%, 95% CI 24.6–43.4) than any other type of brain tumor. In case they were located supratentorially, they involved the frontal lobe more often than any other lobe or subcortical structure (31.2%, 95% CI 22.7–41.2). BMs from NSCLC adenocarcinoma tumors seemed to have a slight preference for the frontal lobe (34.4%, 95% CI 23.7–47). NSCLC SCC metastases involved the occipital lobe (27.8%, 95% CI 12.5–50.9) and especially the visual system more often (22.2%, 95% CI 9–45.2) than any other tumor type. NSCLC SCC metastases have not been observed to occur in any central brain structures such as brain stem, thalamus, and basal ganglia; also no affection of the insula was noted. BMs of SCLC tumors showed the strongest preference for the frontal lobe (51.5% 95% CI 35.2–67.5) but also occurred in subcortical structures [basal ganglia (15.2%, 95% CI 6.7–30.9) but not thalamus]. Among all BMs, they involved the corpus callosum most often (15.2%, 95% CI 6.7–30.9).

### Appearance-Based Magnetic Resonance Features

#### Glioblastoma *Versus* Metastasis

**Figure 5:**
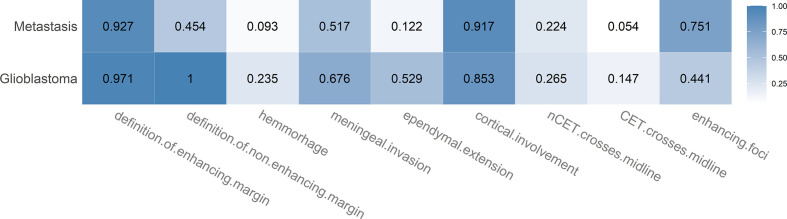
Heatmap showing the proportions for the different appearance-based MR features in case of brain metastases and glioblastoma. Each proportion corresponds to the ratio: number of lesions with feature/total number of lesions of that class. Darker color indicates a larger proportion.

**Figure 5** shows the proportions of appearance-based MR features for BMs and GBMs. When compared to BMs, GBMs exhibited a varied appearance with strong preference for ependymal extension (52.9%, 36.7–68.5 *vs.* 12.2%, 8.4–17.4), hemorrhage (23.5%, 12.4–40 *vs.* 9.3%, 6–14) and slightly for meningeal invasion (67.6%, 50.8–80.9 *vs.* 51.7%, 44.9–58.5). Furthermore, they were a lot less likely to show more than one enhancing focus per patient than BMs were (44.1%, 28.9–60.6 *vs.* 75.1%, 68.8–80.5). The non-enhancing margin of the tumor affected brain tissue, usually corresponding to the outline of the peritumoral edema, was always well-defined in GBMs as opposed to BMs (100%, 89.8–100 *vs.* 45.4%, 38.7–52.2). Interestingly, crossing of the mid-line by enhancing tumor parts was similarly less likely in GBMs than in BMs (14.7%, 6.4–30.1 *vs.* 5.4%, 3–9.4), and the enhancing margin of BMs was no more likely to be well defined than the one of GBMs (92.7%, 88.3–95.5 *vs.* 97.1%, 85.1–99.5).

#### Comparison of Histological Subtypes

**Figure 6:**
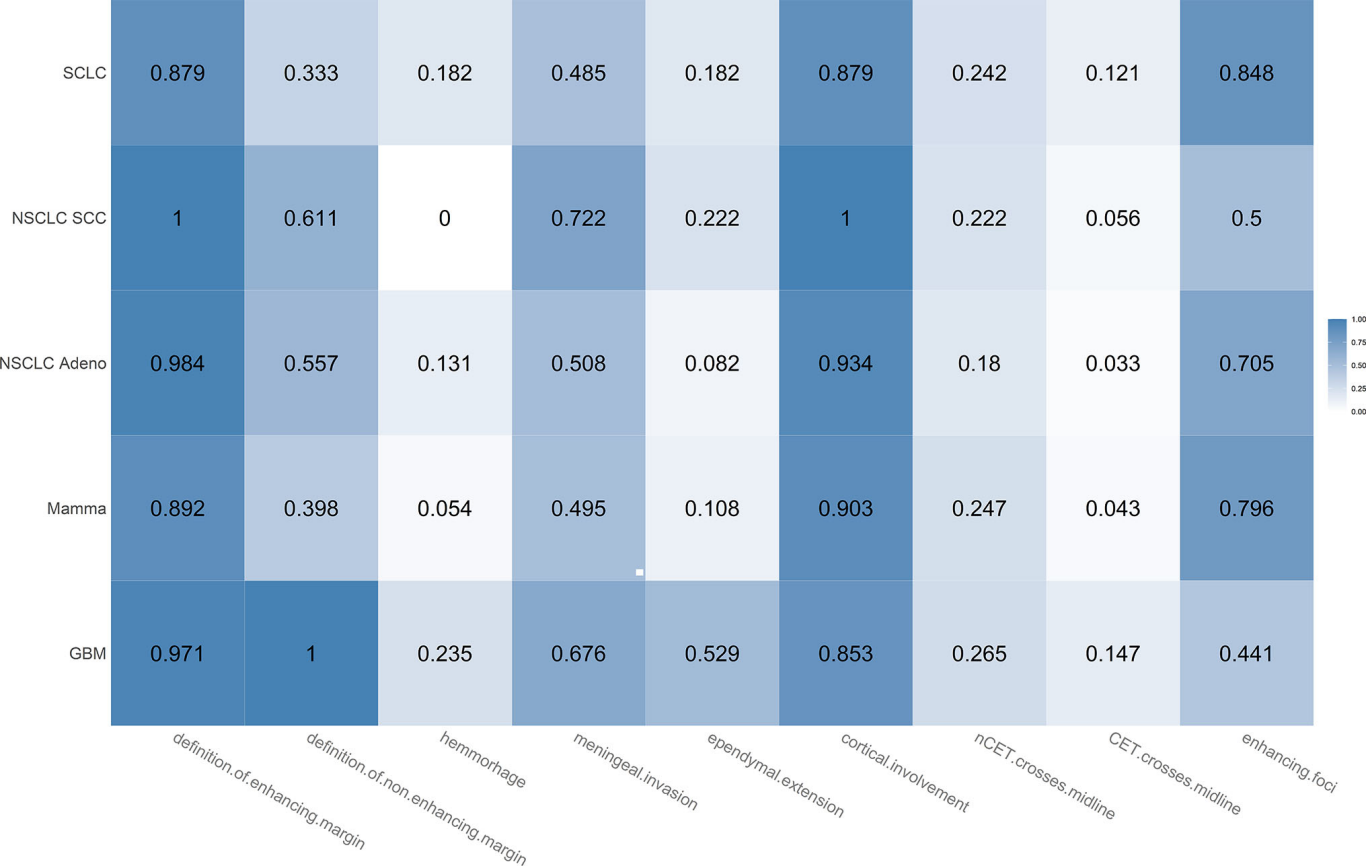
Heatmap showing the proportions for the different appearance-based MR features broken down for the different histological subtypes of brain metastases (SCLC, NSCLC SCC, NSCLC adenocarcinoma, mamma carcinoma). Each proportion corresponds to the ratio: number of lesions with feature/total number of lesions. Darker color indicates a larger proportion.

In **Figure 6**, the proportions of appearance-based MR features are broken down for the different histological subtypes of BMs. In general, the differences in appearance-based MR features among BMs of different histological types seemed to be subtle. BMs from NSCLC SCC tumors were most frequent to show meningeal invasion (72.2%, 95% CI 49.1–87.5) and ependymal extension (22.2%, 95% CI 9–45.2), but they did not exhibit hemorrhage in any evaluated case. SCLC tumor metastases showed most often multiple enhancing foci (84.8%, 95% CI 69.1–93.3).

### Unsupervised Analysis of Combined Magnetic Resonance Features

**Figure 7:**
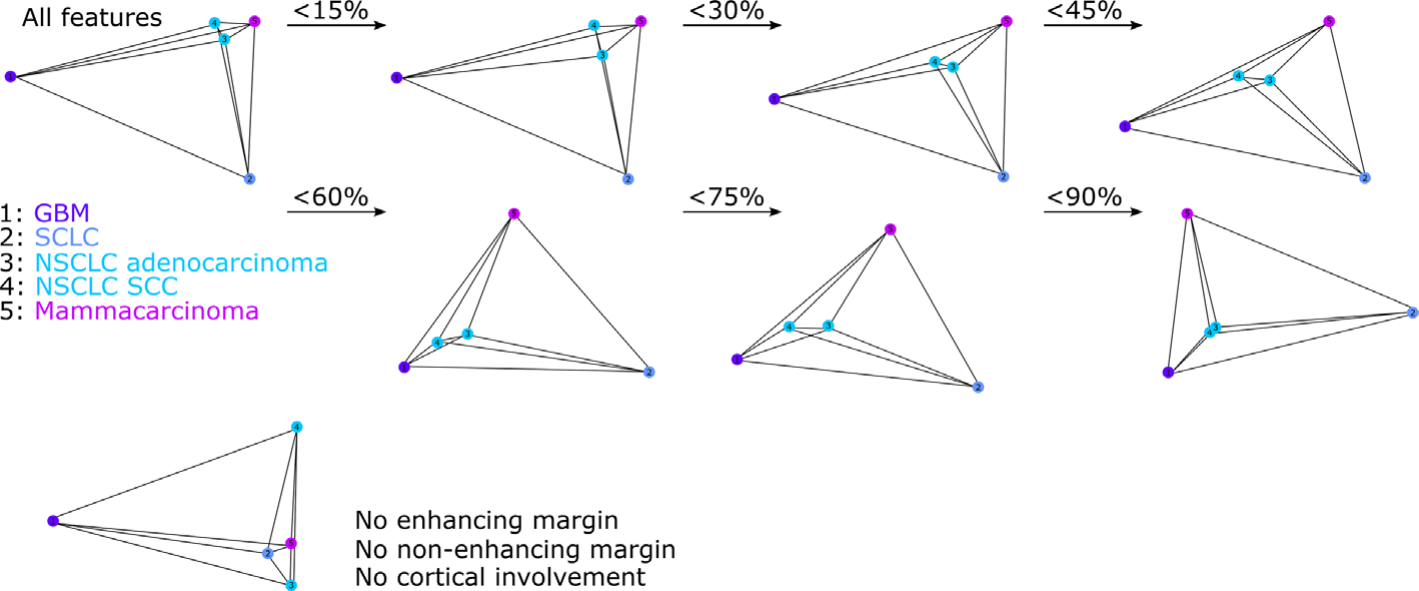
Spectral embedding of the distance matrix for all tumor types and all VASARI features (outer left side); all features but tumor margins and cortical involvement (bottom left), and for different feature exclusion thresholds (<15 to <90%, based on proportions).

Based on the previous results, features were incrementally excluded (in 15% steps) if proportions were below a fixed threshold (<15 to <90% across all histological tumor types). In the case of the <15% threshold, the excluded features included “insular”, “brainstem”, auditory center”, “Wernicke”, “Broca”, “somatomotor”, and “CET crosses midline” (seven features: six localization-based and one appearance-based feature). In **Supplementary Figure 1**, the result of an agglomerative hierarchical clustering of the reduced feature set (for <15% threshold) is shown. The corresponding mean Jaccard distance among the different primary tumors is shown in **Table 2**. The NSCLC SCC BMs exhibited the lowest intra-class Jaccard distance (0.508) among all histological subtypes, which indicates that they appear to be more homogeneous. The distance matrix for a given threshold (*e.g.* <15%) can be transformed to an affinity matrix of an undirected graph and visualized using a spectral layout (**Figure 7**). We can observe that overall GBM and SCLC appear to be very different from the remaining three histological subtypes. The same observation can be made if all available features are used (see **Figure 7**, outer left). With an increasing threshold, the nodes of NSCLC adenocarcinoma and NSCLC SCC move closer towards GBM and away from mammacarcinoma. For the <90% threshold, only three appearance-based features remained: definition of enhancing margin, definition of non-enhancing margin, and cortical involvement. In the case that only those features are excluded, NSCLC SCC and SCLC trade their position when compared to the previous configuration (with NSCLC Adeno, NSCLC SCC, and mammacarcinoma forming a cluster) (*cf*. **Figure 7**, bottom left).

**Table 2:** Mean Jaccard distance summarized for all lesions of a particular tumor type based on binary VASARI feature vectors (using the 15% threshold for exclusion).

	GBM	Breast cancer	NSCLC Adeno	NSCLC SCC	SCLC
**GBM**	0.522	0.651	0.621	0.616	0.640
**Breast cancer**	0.651	0.575	0.583	0.589	0.593
**NSCLC Adeno**	0.621	0.583	0.559	0.579	0.590
**NSCLC SCC**	0.616	0.589	0.579	0.508	0.605
**SCLC**	0.640	0.593	0.590	0.605	0.560

### Supervised Analysis of Combined Magnetic Resonance Features

**Figure 8:**
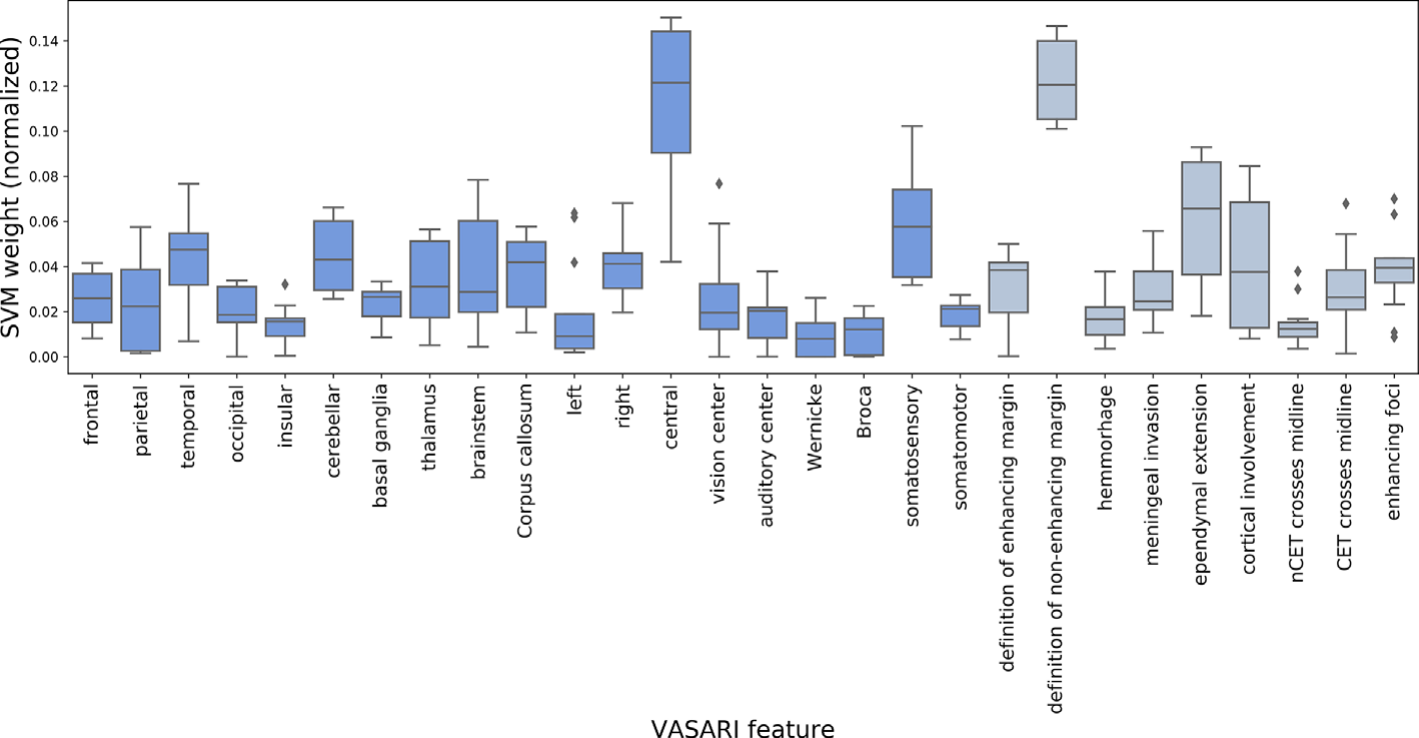
Result for repeated fitting of linear soft-margin Support Vector Machine (SVM) for a total of 12 different hyperparameter configurations. Higher median SVM weights across different hyperparameter configurations indicate VASARI feature which are more important for differentiation of BMs from GBMs (dark blue = localization-based features, gray = appearance-based features).

**Figure 9:**
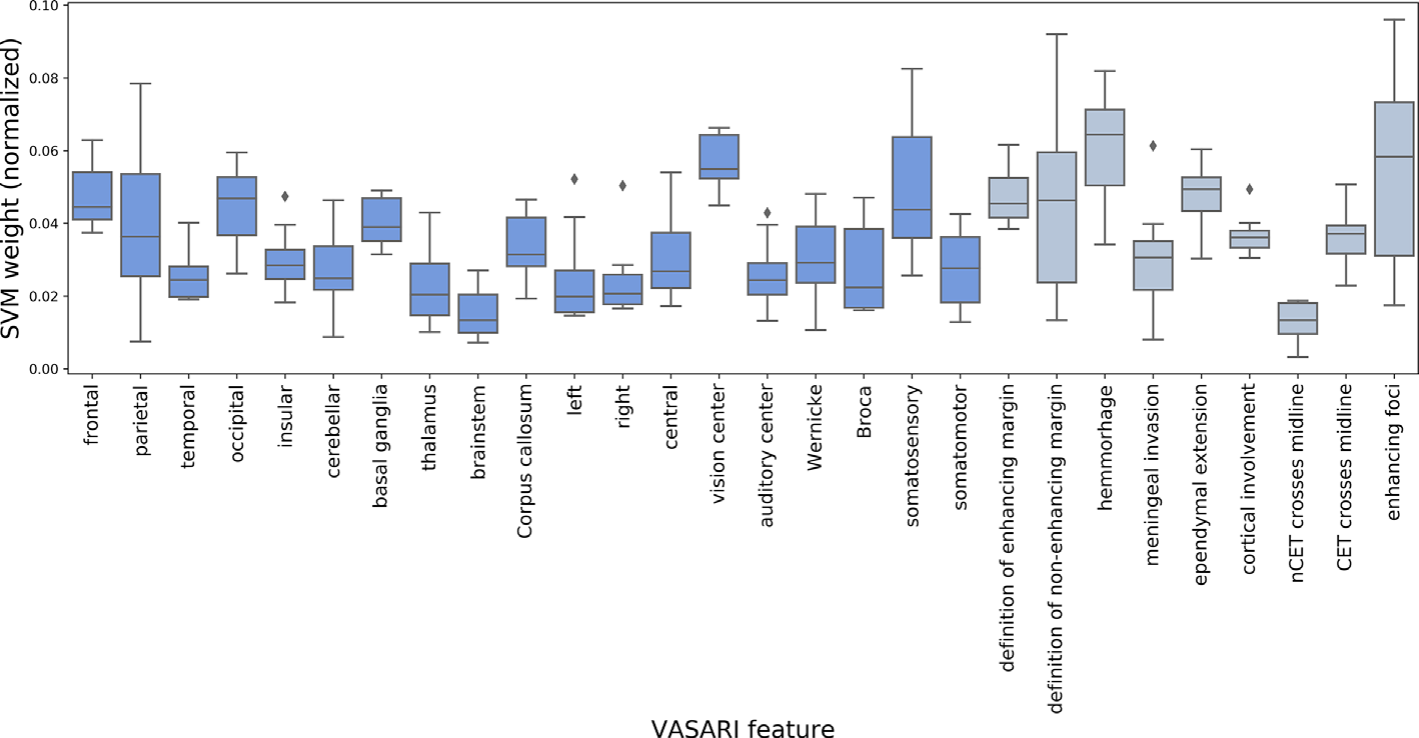
Result for repeated fitting of linear soft-margin Support Vector Machine (SVM) for a total of 12 different hyperparameter configurations. Higher median SVM weights across different hyperparameter configurations indicate VASARI feature which are more important for differentiation of histological BM subtypes (4 classes) (dark blue = localization-based features, gray = appearance-based features).

In **Table 3**, the results of the univariate ranking using mutual information between feature values and target labels are shown alongside the multivariate ranking using either repeated fitting (varied hyperparameters) or recursive feature elimination (RFE, using optimal hyperparameters) based on linear soft-margin Support Vector Machines (SVMs). The features are ranked according to their scores (or weights) from the most important to the least important one. For differentiation of GBMs from BMs, high scores for definition of non-enhancing margin, ependymal extension, and features describing laterality were observed in case of both univariate and multivariate analyses. For differentiation of histological subtypes of BMs, high scores for definition of enhancing and non-enhancing margin and localization in the vision center appeared in case of both univariate and multivariate analyses. In addition, **Figures 8** and **9** show the results of the repeated fitting of the SVM algorithm for different hyperparameter configurations. For differentiation of GBMs from BMs, the central localization and definition of non-enhancing margin were weighted consistently high. A gridsearch based on 3-fold cross-validation using all available features led to an optimal hyperparameter setting (C = 1, no class balancing) with a weighted F1-score of 0.865 for the differentiation of GBMs and BMs and an optimal hyperparameter setting (C = 0.1, with class balancing) for the differentiation of the BM subtypes with a weighted F1-score of 0.326.

**Table 3:** Results of univariate and multivariate feature ranking (repeated fitting or recursive feature elimination) for differentiation of brain metastases (BM) from glioblastoma (GBM) as well as the differentiation of the four different histological BM subtypes (mammacarcinoma, NSCLC adenocarcinoma, NSCLC SCC, SCLC).

Univariate ranking (Mutual information BM vs. GBM)	Multivariate ranking (linear soft-margin SVM, BM vs. GBM, median weight for 12 models)	Multivariate ranking (RFE, linear soft-margin SVM, BM vs. GBM, C=1, no class balancing)
**definition of non-enhancing margin** enhancing foci **left** **ependymal extension** vision center **central** CET crosses midline temporal occipital thalamus Broca parietal cerebellar insular Corpus callosum definition of enhancing margin basal ganglia Wernicke frontal brainstem right somatosensory somatomotor nCET crosses midline meningeal invasion hemmorhage cortical involvement auditory center	**Central** **definition of non-enhancing margin** **ependymal extension** somatosensory temporal cerebellar Corpus callosum **right** enhancing foci definition of enhancing margin cortical involvement thalamus brainstem basal ganglia CET crosses midline frontal meningeal invasion parietal somatomotor auditory center vision center occipital hemmorhage insular nCET crosses midline Broca left Wernicke	**definition of non-enhancing margin** **central** **ependymal extension** **right** temporal frontal occipital somatosensory parietal brainstem cortical involvement auditory center enhancing foci somatomotor basal ganglia nCET crosses midline CET crosses midline cerebellar meningeal invasion Corpus callosum hemmorhage definition of enhancing margin vision center thalamus Broca left insular Wernicke
**Univariate ranking (Mutual information, multi-class, histological subtypes of BMs**)	**Multivariate ranking (linear soft-margin SVM, multi-class, histological subtypes of BMs, median weight for 12 models)**	**Multivariate ranking (RFE, linear soft-margin SVM, multi-class**, **histological subtypes of BMs**, **C=0.1, with class balancing)**.
basal ganglia **vision center** nCET crosses midline meningeal invasion **definition of non-enhancing margin** **definition of enhancing margin** cortical involvement insular somatosensory cerebellar CET crosses midline temporal auditory center right occipital brainstem parietal somatomotor thalamus left hemmorhage frontal ependymal extension enhancing foci central Wernicke Corpus callosum Broca	hemorrhage enhancing foci **vision center** ependymal extension occipital **definition of non-enhancing margin** **definition of enhancing margin** frontal somatosensory basal ganglia CET crosses midline parietal cortical involvement Corpus callosum meningeal invasion Wernicke insular somatomotor central cerebellar temporal auditory center Broca right thalamus left nCET crosses midline brainstem	enhancing foci **definition of non-enhancing margin** frontal **vision center** hemmorhage meningeal invasion left **definition of enhancing margin** ependymal extension occipital somatosensory insular cerebellar cortical involvement basal ganglia temporal right somatomotor CET crosses midline Wernicke central parietal auditory center Broca Corpus callosum nCET crosses midline thalamus brainstem

For the multi-class problem, the Support Vector Machine (SVM) was trained in a one-vs-all fashion (bold, black font = features which are consistently top-ranked). The former corresponds to a binary classification problem, whereas the latter is a multi-class classification problem (four classes).

## Discussion

In this study, we investigated the potential of the VASARI MR feature guide in differentiating BMs of different primaries from GBMs using localization-based and appearance-based features. The VASARI MR feature guide has been developed for primary brain tumors and has mostly been applied to GBMs (13, 18–24) and in a few studies to lower grade gliomas (26–28). To the best of our knowledge, this is the first study applying VASARI features to BMs. An explorative approach was chosen as a first evaluation of applicability of VASARI features in this setting.

We found that GBMs differ from BMs both (i) in their preferred localization and (ii) MR image appearance: i.) GBMs involved all lobes of the cerebrum with slight fronto-occipital gradient, often affected central structures and showed a strong preference for the right hemisphere, whereas BMs occurred most frequently in the frontal lobe and in the cerebellum. ii.) GBMs always exhibited a well-defined non-enhancing margin and appeared more often than BMs as solitary lesions and/or with ependymal extension. Differences among BMs were very subtle; the only distinct finding was that NSCLC SCC metastases were localized occipitally affecting the vision center more often than any other type of BM.

Unsupervised analysis of combined MR features showed that NSCLC adenocarcinoma and NSCLC SCC BMs appear to be most similar and that this similarity is driven by the definition of tumor margins and cortical involvement. Furthermore, GBM appear to be distinctively different from all types of BMs for different feature subsets. Supervised analysis of combined MR features showed for differentiation of GBMs from BMs high scores for definition of non-enhancing margin, ependymal extension, and features describing laterality in case of both univariate and multivariate analyses. For differentiation of histological subtypes of BMs, high scores for definition of enhancing and non-enhancing margin as well as localization in the vision center appeared in the case of both univariate and multivariate analyses. A gridsearch based on 3-fold cross-validation using all features yielded an optimal model with weighted F1-score of 0.865 for the differentiation of GBMs from BMs. For the differentiation of all tumor types, the optimal model led to a weighted F1-score of 0.326.

In the spirit of multiverse analysis (34), *i.e.* by viewing the data from different statistical angles, the same features and “feature families” have come up repeatedly, substantiating suspicion that these might play a role in brain tumor differentiation: i.) definition of tumor margins, ii.) ependymal extension, iii.) tumor localization including the involvement of subcortical gray matter structures and laterality.

- non-enhancing tumor margin: GBMs presented with well-defined non-enhancing tumor margin more often than BMs. Previously, brain lesions have been found to show peritumoral edema if they are larger than ~9.5 mm in diameter (35). GBMs are usually large at the time of diagnosis, thus typically exhibiting extensive edema with a well-defined margin. The extent of edema is constrained by the surrounding gray matter structures (*e.g.* cortex), which may also contribute to the increased perception of the edema’s definedness. In our population, some BMs were very small and did not exhibit any peritumoral edema; therefore we argue that they also did not present with a well-defined non-enhancing tumor margin. In a future study, we plan to investigate the relationship between the volume of edema and its radiological presentation. Furthermore, qualitative differences in T2-weighted signal alterations between high-grade gliomas and BMs have been demonstrated previously with high-grade gliomas exhibiting more frequently high signal intensity of the cortex for non-enhancing tumor regions on T2-weighted FLAIR sequences when compared to brain metastases (36). A third aspect might be image quality. As for the GBMs in our study a standardized imaging protocol was used; MR images of BMs were acquired with a variety of different protocols. Consequently, the comparability of the MR images suffered.
- ependymal extension: The dogma of the brain being a quiescent organ without neuronal regenerative potential is outdated. Multipotent, self-renewing neural stem cell populations have been confirmed in the forebrain subventricular zone (SVZ) and the subgranular zone (SGZ) of the dentate gyrus. These populations are capable of neurogenesis in the adult brain, and it is widely acknowledged that glioma initiating cells arise from these populations (37–40). Because of the close spatial proximity of the SVZ to the lateral ventricles, the feature “ependymal extension” might be viewed as a surrogate marker for the involvement of the SVZ. This potentially explains why ependymal extension was more often present in GBMs than in BMs which emerge in the brain through hematogenic spread of systemic tumor cells.
- localization: It is still unclear if and why BMs preferably arise in certain localization of the brain. The most accepted hypothesis is that the rate of metastases is proportional to the blood flow in this area (41, 42). This hypothesis is in accordance with our findings of bigger cerebral lobes exhibiting more metastases. Moreover, BMs from breast cancer seemed to have particular affinity to the cerebellum, which has also previously been described in the literature (43). Compared to BMs, GBMs occurred more often in subcortical gray matter structures such as the basal ganglia, involved the corpus callosum and presented with a slight decreasing fronto-occipital gradient. This finding has previously been described by Larjavaara et al. (44). whose “findings indicate that gliomas arise mainly from the anterior subcortical structures of the brain, with an excess in the frontal and temporal lobes that is not accounted for by tissue volume alone.” This can be explained by the close spatial proximity of the previously discussed origin of glioma initiating cells in the SVZ and SGZ. Furthermore, radiographic atlases of GBMs showed high tumor incidence in periventricular white matter regions and found that laterality and involvement of the frontal lobe may be related to underlying genetic and molecular characteristics of the tumor (45).

In general, our results could be useful in defining a subset of MR features that help radiologists to differentiate between GBMs and BMs in a more structured manner. As especially GBMs exhibit some distinct characteristics, it could be argued that in the absence of these, BM becomes the more likely diagnosis. Based on our observations, the differentiation of GBMs from BMs using definition of non-enhancing margin, ependymal extension and localization in the brain should be evaluated as imaging biomarkers for differential diagnosis in an independent confirmatory analysis.

Some limitations of the study should be noted. Despite the extensive inclusion period (years 2000 through 2018), we obtained only small sample sizes for rare types of BMs (NSCLC SCC and SCLC with 13 and 11 patients, respectively) which caused a class imbalance when compared to more frequently occurring BMs (NSCLC adenocarcinoma and mamma carcinoma with 30 included patients each). Aware of this imbalance, we intended to compensate by applying statistical techniques that account for different group sizes: i) we used stratified cross-validation in order to ensure that the original class distribution is maintained, ii) we use class-weighting as a hyperparameter for the SVM to adapt it for handling imbalanced classes, and iii) we use the weighted F1-score as performance metric, which is computed for each class label independently and weighted by its support thus providing a robust classification metric for imbalanced data. Since the initial class distribution of the four tumor types approximates their prevalence in clinical routine and the class distribution is maintained throughout our analysis, we obtain an estimation of the SVMs’ classification performance in a setting which closely reflects the clinical scenario. With the extensive inclusion period another issue arose: the heterogeneity of imaging protocols and image quality. At our institution, a standardized imaging protocol for brain tumor patients exists since 2014. Therefore, available images and image quality varied widely over the years. This can potentially lead to failure to detect the smallest lesions or slight alteration of lesion features. On the contrary, one can argue that the features which were found most discriminative are so in a manner robust to different image acquisition protocols.

The applied VASARI features were capable of effectively highlighting differences between GBMs and BMs, which were reflected in descriptive statistics, showing consistently large inter-tumoral distances in unsupervised analyses, and high weighted F1-score for binary discrimination in supervised multivariate analyses. Definition of non-enhancing margin, ependymal extension, and tumor localization seem to play a major role. Regarding the differentiation between histological types of BMs, differences were much less accentuated. They seem to be driven mainly by definition of tumor margins, and localization in the vision center.

## Data Availability Statement

The raw data supporting the conclusions of this article will be made available by the authors, without undue reservation.

## Ethics Statement

The studies involving human participants were reviewed and approved by Kantonale Ethikkomission Bern. Written informed consent for participation was not required for this study in accordance with the national legislation and the institutional requirements.

## Author Contributions

RM and UK designed the experiments. AP and UK rated and prepared the data. RM and AP performed the statistical data analysis. RM, AP, RW, and UK interpreted the results. RM and AP drafted the manuscript. RW and UK revised the manuscript. All authors contributed to the article and approved the submitted version.

## Funding

This study was supported by funding received from Swiss Personalized Health Network (SPHN, project number 2018DRI10).

## Conflict of Interest

The authors declare that the research was conducted in the absence of any commercial or financial relationships that could be construed as a potential conflict of interest.
