# Supplementary material for: Exploratory Analysis of Qualitative MR Imaging Features for the Differentiation of Glioblastoma and Brain Metastases
Source: Front Oncol. 2020 Dec 10;10:581037. doi: 10.3389/fonc.2020.581037 (PMC7793795; doi:10.3389/fonc.2020.581037)
Supplement: Supplementary file 1 [file Image_1.pdf]

Supplementary Material

1.1 Supplementary Figures

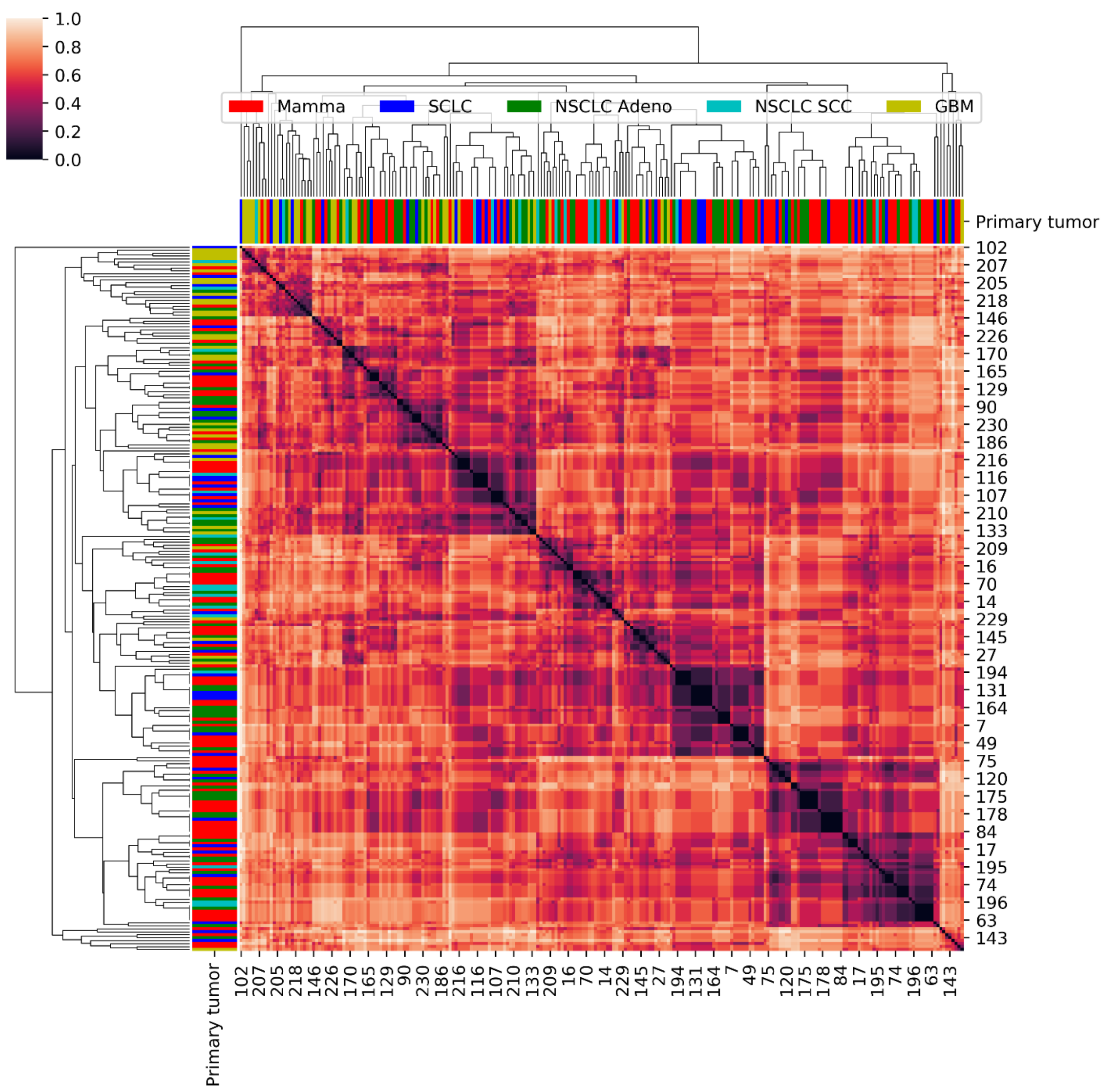

Supplementary Figure 1. Result of an agglomerative hierarchical clustering based on Jaccard distance between morphology of lesions described with binary VASARI feature vectors (using the 15% threshold for exclusion).
